# Going home with baby: innovative and comprehensive support for new mothers

**DOI:** 10.1017/S1463423618000932

**Published:** 2018-12-27

**Authors:** Tonia Olson, Angela Bowen, Julie Smith-Fehr, Swagata Ghosh

**Affiliations:** 1 Clinical Coordinator, Healthy & Home, West Winds Primary Health Centre, Saskatchewan Health Authority, Saskatoon, SK, Canada; 2 Professor, College of Nursing, University of Saskatchewan. Saskatoon, SK, Canada; 3 Maternal Services Manager Healthy & Home/Prenatal Home Care/Baby-Friendly Initiative Coordinator, West Winds Primary Health Centre, Saskatchewan Health Authority, Saskatoon, SK, Canada; 4 Research and Statistical Officer, Department of Health and Wellness, Government of Nova Scotia, Halifax, Canada

**Keywords:** baby-friendly initiative, breastfeeding, community-based support for mothers, postpartum care, primary health care, safe postpartum maternal and newborn discharge

## Abstract

Shorter length of stay for postpartum mothers and their newborns necessitates careful community follow-up after hospital discharge. The vast amount of information given during the initial postpartum period can be overwhelming. New parents often need considerable support to understand the nuances of newborn care including newborn feeding. Primary health care and community services need to ensure there is a seamless continuum of care to support, empower, and educate new mothers and their families to prevent unnecessary hospital readmission and other negative health outcomes. The Healthy & Home postpartum community nursing program provides clinical communication and supports to bridge the gap between acute hospital and community follow-up care through home visits, a primary health care clinic, a breastfeeding center, a breastfeeding café, a postpartum anxiety and depression support group, bereavement support, and involvement in a Baby-Friendly Initiative™ coalition. Nurses working in the program have the acute care skills and resources to complete required health care assessments and screening tests. They are also international board-certified lactation consultants able to provide expert breastfeeding and lactation care. This paper describes how the Healthy & Home program has evolved over the past 25 years and offers suggestions to other organizations wanting to develop a postpartum program to meet the physical and mental health needs of postpartum families to promote maternal and infant wellbeing.

Childbirth is recognized as a time of significant physiological adaptation for both the mother and newborn, but it is also a period of considerable psychological and psychosocial adjustment, which extends to families and communities (Chalmers *et al.*, 2008; Phillips *et al.*, 2013; Haran *et al.*, 2014). In a systematic review of postpartum clinical guidelines from Australia, the United Kingdom, and the United States, Haran *et al.* (2014) reported that while many women transition through this time without concern, others develop significant health issues. Hemorrhage, fever, abdominal and back pain, urinary tract complications, and thromboembolitic disease have been observed (Bashour, 2008). Perineal and cesarean section wound, breast, and vaginal pain are also reported (Chalmers *et al.*, 2008; Declerq *et al.*, 2008). Breastfeeding difficulties are common (Chaput *et al.*, 2016) and rates of postpartum depression and anxiety are known to be as high as 15–20% in developed countries (WHO, 2016). For newborns, respiratory distress, temperature instability, and jaundice are common clinical problems (Wang *et al.*, 2004).

The increasing acuity of mothers and newborns in hospital coupled with the trend of early discharge presents a growing challenge for health care administrators and policymakers to ensure access to timely and appropriate community follow-up, particularly for those in more complex medical and social situations. This includes support after discharge for feeding of the late preterm newborn, which is often more complicated as the newborn may be sleepier and may have less stamina than a term newborn to coordinate and maintain effective suck–swallow patterns (The Academy of Breastfeeding Medicine, 2011). Moreover, while preterm newborns may be responsive to the benefits of early discharge, such as improved infant feeding, increased parental confidence, and increased stability of the family unit (Whyte, 2010), these newborns are also more susceptible to the risks of going home early, such as readmission due to hyperbilirubinemia, poor feeding, infection, respiratory problems, and apnea (Engle *et al.*, 2007; Phillips *et al.*, 2013).

In developed countries, the length of hospital stay after childbirth has fallen dramatically in the last 30 years and it is expected that postpartum health care services are of the highest standard (Brown *et al.*, 2002). Early postpartum follow-up in the community can lead to early identification and resolution of maternal–newborn illness, and may prevent any health problems of mom and baby from becoming chronic (Yonemoto *et al.*, 2017). Postpartum home visits also improve practices such as skin-to-skin contact, initiation and maintenance of breastfeeding, and delayed bathing, and allow for timely assessment of maternal mental health, family circumstances, and environmental concerns, which may impact the newborn’s health and welfare (Yonemoto *et al.*, 2017). This article describes how a community postpartum nursing program located within a primary health care center, Healthy & Home, in Saskatchewan, Canada, has effectively evolved to meet the acute care needs of postpartum families and how it promotes maternal and infant wellbeing through its various components. A librarian-led review of the published and gray literature was conducted to identify similar maternity programs, with none located.

## Healthy & Home program

The Healthy & Home program provides a continuum of services including a home visiting program, breastfeeding centers, a breastfeeding café, a clinic, a postpartum anxiety and depression support group, and telephone bereavement support to bridge care between early discharge from acute maternity care and the many needs of mothers as they adapt to motherhood and care for their newborns at home. The program operates under the auspices of Maternal Services of the Royal University Hospital: a tertiary acute care facility with over 5600 live births annually. Healthy & Home is strategically located within an easily accessible primary health care center to maintain a community, family-friendly focus.

When the Healthy & Home program began in 1992, home visits by a nurse were offered to mothers who had given birth to healthy, term newborns discharged from hospital ‘early’ (meaning earlier than two days post vaginal or four days post cesarean birth). Home visits continued, as needed, until the newborn was three days old if born vaginally, or five days old if born by cesarean section. In the first year, 1014 families received care, with a total of 1245 appointments completed. Since that time, in response to the trend of earlier discharge along with recommendations for postpartum surveillance of conditions including gestational hypertension (Magee *et al.*, 2008) and newborn hyperbilirubinemia (Barrington and Sankaran, 2007), Healthy & Home has expanded to now serve over 4000 families through over 7100 appointments and telephone-based bereavement support to approximately 50 clients a year. Our mandate now is to offer services within 24–48 h of hospital discharge to *all* mother–newborn dyads residing in the catchment area regardless of hospital stay and/or acuity level. Increasingly, earlier discharge and growing acuity at discharge have necessitated that Healthy & Home now follows many families well beyond postpartum day five either in the home, or in a clinic setting for dyads who are stable and able to commute. Infants discharged from neonatal intensive care or the pediatric unit receive follow-up care if sent home prior to eight days of life.

**Figure 1:**
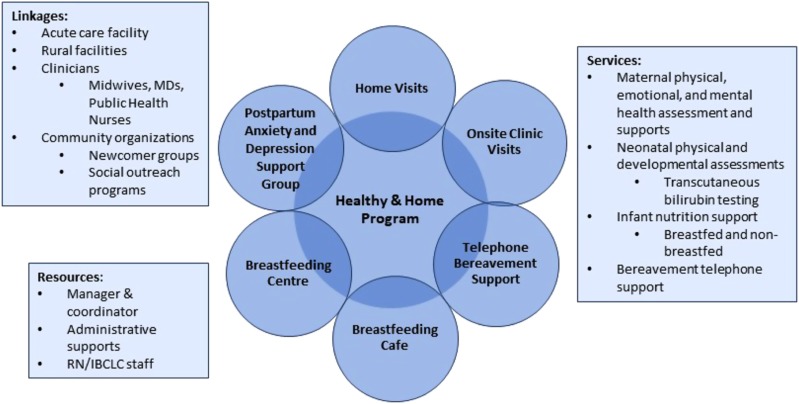
Healthy & Home program components

The program is staffed by a manager, a clinical coordinator, 15 permanent part-time registered nurses with the international board-certified lactation consultant (IBCLC) credential, and three part-time administrative assistants. A registered nurse/IBCLC is assigned to manage a phoneline for clients and clinicians daily, seven days a week year-round. See Figure 1 for the program components.

Upon discharge from hospital, Healthy & Home receives maternal contact information, as well as pertinent labor, birth, and postpartum assessment information via scanned email on an encrypted secure system. A Healthy & Home nurse then triages to determine whether a home visit is warranted within 24 h or whether the family would be better served with a day of rest and receive a follow-up phone call or visit within 48 h (Maternal Fetal Medicine Committee of the Society of Obstetricians and Gynaecologists of Canada, 1996; Whyte, 2010). While all mothers are contacted by phone, around 14% a year opt for ongoing phone support rather than a home visit; most of these are women who have received Healthy & Home services in the past and are comfortable with newborn care and accessing resources such as their primary health care provider, breastfeeding center, or mental health supports as needed.

Healthy & Home serves as a resource for both pregnant and postpartum women and for other health care professionals in the region. Family medicine and pediatric medical residents, psychiatric residents, and nursing students can spend time in the breastfeeding center, clinic, the postpartum anxiety and depression group or attend home visits as part of their clinical rotations.

## Home visits

Home visits are done from 8:30 am to 4:30 pm seven days a week. The nurse performs physical assessments of the mother and infant while considering any mental health, social, and environmental factors, which might affect family adaptation. Maternal assessment includes vital signs, breast and nipple condition, fundal height and lochia, perineum, wound area (post cesarean section staples removal if necessary), diet, family support, and risk for postpartum anxiety and depression. Infant assessment includes vital signs, reflexes, skin condition, fontanelles, output patterns, and weight. Abnormal findings are reported to the primary health care provider. Home visits are scheduled around newborn feeding times whenever possible to facilitate feeding assessments, appropriate support, and follow-up. Transcutaneous (TCB) screening for newborn hyperbilirubinemia is done to reduce heel pokes. TCB screening is part of a unique program that uses a locally validated predictive TCB nomogram (McClean *et al.*, 2018). Any required newborn blood work, such as the metabolic screen, total serum bilirubin, and direct antiglobulin test are drawn and taken to the laboratory by the nurse.

At the end of the initial home visit, the nurse in consultation with the family formulates a plan of care, which may include additional home visits, a clinic appointment, telephone follow-up and/or follow-up at a breastfeeding center or referral to other health care practitioners. There is no standardized endpoint to the services a family will receive from Healthy & Home; rather, the goal of care is to achieve stability, safety, and support, which considers the family’s perception of need and any cultural and/or psychosocial considerations. Any outstanding medical concerns are communicated to the most responsible physician or midwife for timely follow-up. If no follow-up is required, pertinent community supports are discussed including the peer-to-peer breastfeeding café support group, the local Mother’s Centre, which also provides peer-based breastfeeding support, or the local La Leche League group (www.lllc.ca, 2018). The family’s referral information is then passed on to the public health office and the family is advised to have their newborn assessed by their family physician in 1–2 weeks for ongoing care.

## Breastfeeding center

Early in Healthy & Home’s development, the importance of consistent, evidenced-based care for mothers wishing to breastfeed was recognized. The decision was made that all permanent staff must have IBCLC designation in order to optimally support families to meet their infant feeding goals and to prevent health care concerns often related to poor feeding such as excessive weight loss, dehydration, hyperbilirubinemia, and failure to thrive. The broader significance of breastfeeding support, such as improved long-term health outcomes, is widely established in the literature (Bartick and Reinhold, 2010; Duijts *et al.*, 2010; McNiel, Labbok and Abrahams, 2010; Renfrew *et al.*, 2012).

A 1993 program evaluation found that 85% of the phone calls to Healthy & Home were related to breastfeeding (Khachatourians, 1993); therefore, our first breastfeeding center was opened in 1994. The purpose of the center was to increase maternal knowledge, skill, confidence, and motivation to breastfeed with a long-term goal to meet the World Health Organization’s recommendation for optimal infant nutrition of newborns for six months of age (Stefiuk *et al.*, 2002). Families may self-refer, or may be referred by a health care provider for individual appointments, and/or telephone consultation available Monday–Friday from 8:30 am to 4:30 pm. To increase the accessibility of breastfeeding services, drop-in satellite sites were established in three local shopping malls. These satellite sites provided a place for mothers to weigh their newborns and to receive informal support from a lactation consultant and other mothers. Today we have two breastfeeding centers, with the second one opened in 2014 as part of a primary health care center within a family medicine practice, which contains a human milk drop.

## Breastfeeding Café

We started a drop-in breastfeeding café in 2010 within our primary health center. The café concept (a relaxed atmosphere where mothers can access breastfeeding information and support from a lactation consultant and other mothers) was initiated to encourage the vital role peer support plays in helping mothers to meet their breastfeeding goals. The group format is informal, with an emphasis on open discussion. Guest speakers are invited if mothers request information on topics related to infant feeding such as newborn massage, newborn wearing, food allergies, and starting solids. In an evaluation during 2013, women reported that the café format enhanced their breastfeeding and parenting experience and that they appreciated the opportunity to have their questions answered, to relate, and to learn from other mothers. Connections made in the café often last well beyond the time spent in the group, with some women going on to start their own toddler support, walking, and social media groups.

## Baby-Friendly™ designation

With the efforts and initiatives of Healthy & Home, the primary health center achieved Baby-Friendly™ designation in 2011 and again in 2016. The goal of the Baby-Friendly Initiative (BFI) is to improve the rates of breastfeeding initiation and duration (World Health Organization, 2009). After achieving BFI designation, Healthy & Home along with Breastfeeding Matters (a local grassroots mother’s organization) and the Child Hunger and Education Program of Saskatoon, rejuvenated a local Baby-Friendly™ coalition to address specific barriers in the promotion and support of the Baby Friendly Ten Steps (Breastfeeding Committee for Canada, 2017). The coalition promotes capacity building, staff development, and public education, while bolstering community partnerships to improve maternal–child health outcomes.

## Postpartum anxiety and depression support group

Maternal mental health issues, including postpartum depression and anxiety undermine a mother’s capacity and confidence to care for herself and her newborn (Avis and Bowen, 2004). Access to timely and appropriate breastfeeding support has been shown to be protective of maternal mental health (Chaput *et al.*, 2016). As a result of a needs assessment (Irwin, 1998), Healthy & Home, along with stakeholders from the mental health community in the region, launched a postpartum depression support group. In 2014, the name of the group was changed to acknowledge and raise awareness of the significant prevalence of postpartum anxiety and its effects on maternal and newborn outcomes. Paul *et al.* (2013) found that anxiety was in fact more common than depression in the six months after childbirth and was associated with reduced breastfeeding duration. The group continues to offer mothers a safe place to learn, share, and connect with resources in the community. It meets weekly and is co-facilitated by a mental health professional and a Healthy & Home nurse. Intake assessments are completed by mental health intake workers and include screening with the Edinburgh Postnatal Depression Questionnaire (EPDS) (Cox, Holden and Sagovsky, 1987). The EPDS score is calculated at intake to the program and after discharge. A mental health nurse connects with all participants by telephone before final discharge to ensure that the mother is coping well and that appropriate supports are in place. Referrals to other services (eg, mental health services, psychiatry, and family physician) are made as necessary.

If there are concerns about family violence, trauma, abuse, or addiction, individual counseling and medical supports are suggested along with the information for local shelter supports if needed. Recognizing the challenges of maternal mental illness on concentration and coping, taxis are funded to promote attendance. The group has an informal atmosphere with professional childcare provided free in the room next door, not only to give mothers time to focus on their own needs and healing but also to attend to their newborn for feeding and soothing.

In 2017, 71 referrals were made to the group and a total of 45 group sessions were held with an average of ten women per session. Mothers attend between 2 and 12 sessions. Women were asked to complete the EPDS upon discharge to ensure that they have felt improvement in their mood and coping, and that there are no ongoing feelings of self-harm or harm to others, and to ensure that appropriate referrals have been made if further supports are needed.

## Clinic

To meet the growing demand for services, we established an onsite postpartum clinic within the primary health center in 2014. The clinic setting works well for follow-up visits (eg, to assess feeding, check the newborn’s weight, bilirubin screening, and maternal health). Families with complex breastfeeding needs are referred to the breastfeeding center. While some mothers absolutely cannot or will not travel to visit the onsite clinic for postpartum checks, newborn weights, and feeding support due to their health status, cultural beliefs, or other considerations, other mothers enjoy this opportunity to ‘get out of the house’ with their new newborn.

## Bereavement support

Women who have suffered prenatal loss are at risk for anxiety and depression, which may persist into a subsequent pregnancy and birth of a healthy newborn (Blackmore *et al.*, 2011). Early recognition and intervention may be able to offset the risk of anxiety and depression for these women (Blackmore *et al.*, 2011). Healthy & Home provides follow-up phone support to mothers who have experienced a miscarriage, stillbirth, or neonatal death if the mother wishes. The initial telephone call is made within 1–2 days after hospital discharge, and assessments include both physical and emotional aspects of the client’s recovery. Further telephone follow-up is offered at two weeks. If the nurse has concerns during this call, she will ask the client if they are open to follow-up again at six weeks. Referrals to other supports and services are made as appropriate.

## Evaluation of Healthy & Home (2016–17)

Since 2014, Healthy & Home has used lean methodology to engage staff to identify, test, and implement continuous quality improvement projects (Black and Miller, 2008). Visual management tools now allow for ongoing assessment to optimize client flow and reduce waste. Data are used to monitor, understand, and sustain progress (D’Andreamatteo *et al.*, 2015). Part of this lean work has been to engage in regular self-reflection as a team to set goals for development. For the fiscal year 2016–17, 634 women were invited during their home visit to participate in an online survey to evaluate maternal satisfaction with Healthy & Home services. Only women able to understand and read English, with a working email address, were invited to participate in the online survey format. Those who were very stressed during the visit were not asked to participate. Program evaluations do not require ethical approval within our region; however, women were assured that care would not be affected by a lack of participation.

### Survey instrument

The questionnaire consisted of 17 questions about various aspects of the Healthy & Home program, each with a four-point rating scale ranging from ‘strongly disagree’ to ‘strongly agree’. Respondents were also able to provide any additional comments that they wished to share. Demographic questions included the age of the respondents, the number of children, and the number of Healthy & Home visits they had received.

### Data collection

*The questionnaire was uploaded* to an electronic survey hosting website, and participants in the program were sent a link via email to the survey. Respondents were able to complete the survey at their convenience.

### Data analysis

Data were downloaded from the website into an Excel spreadsheet, cleaned, and collated. Data were transferred to SPSS for quantitative analysis, including frequency counts and variance analysis. Content analysis was used to qualitatively analyze the additional comments provided by the respondents.

### Response rates

Of the 634 mothers, there were a total of 429 responses to the survey, 68% (*n* = 429). There were 26 incomplete responses. These 26 responses were removed from consideration, resulting in 403 useable survey responses.

### Demographics

- 54% were between 30 and 39 years of age
- 96% were partnered or married
- 54% had their first child
- 39% received one visit
- 58% had no previous visits from Healthy & Home nurses
- 24% indicated that they had attended clinic for follow-up.

Of these (*n* = 95), 82% were very satisfied with their experience.

### Survey results

Overall, the mothers are very satisfied with their Healthy & Home experience, with over 96% of mothers agreeing or strongly agreeing that the nurses:

- answered their questions and addressed their concerns;
- visited adequate number of times;
- supported their feeding plan; and
- left them feeling comfortable in their ability to care for their newborn.

## Recommendations to establish a postpartum community program

Those who wish to develop a community postpartum program can learn from our experience. We recommend the following suggestions:

- Collaborate with hospital and community stakeholders. Initially, a hospital liaison nurse may be useful to introduce families, hospital staff, and physicians to the concept of community follow-up following discharge;
- Ensure appropriate and timely community follow-up (eg, make telephone contact with families within 24 h of hospital discharge);
- Ensure nurses have a strong clinical background in acute maternal and newborn care and build confidence to work autonomously in a community setting;
- Make IBCLC designation a requirement for nurses;
- Provide ongoing staff education around maternal and newborn assessments, standards of care, infant nutrition, lactation/breastfeeding, postpartum anxiety and depression, and bereavement;
- Implement the Baby-Friendly Initiative™ in both hospital and primary health care centers; and
- Create a Baby-Friendly coalition, to bring together hospital and community stakeholders such as public health nutritionists, speech language pathologists, mental health workers, community agencies, and grassroots women’s support groups.
- Seek out and maintain acute care and community-based partnerships.

## Conclusion

For 25 years, Healthy & Home has provided comprehensive maternal and newborn assessment and care in a community setting, and now also in primary health centers through phone support, home visits, clinic appointments, and group support. Since the program’s inception, considerable growth has been required to meet demands brought upon by shortened maternity hospital stays, increasing acuity, and the trend toward Baby-Friendly™ standards of care; all of them operating within existing financial resources. The strength of the program is its responsiveness to adapt to the latest best practices and to constantly improve and transform to meet all aspects of postpartum and newborn care, which includes not just physical care of mother and infant feeding but also maternal mental health and bereavement.
